# Intranasal Dexmedetomidine as a rescue strategy for established postoperative delirium following loss of intravenous access: a case report

**DOI:** 10.3389/fmed.2026.1893834

**Published:** 2026-07-07

**Authors:** Yingao Peng, Mingjin Liu, Jiafang Wang

**Affiliations:** 1 Faculty of Medicine, Jianghan University, Wuhan, China; 2 Department of Anesthesiology, Wuhan No.1 Hospital, Wuhan, China

**Keywords:** agitation, dexmedetomidine, elderly, intranasal administration, postoperative delirium

## Abstract

The clinical management of established postoperative delirium (POD) is particularly challenging in patients who exhibit agitated behavior and self-remove intravenous (IV) access. A 70-year-old man with prior cerebral infarction developed POD following urologic surgery under general anesthesia. Two doses of IV diazepam failed to provide sustained relief, and the patient subsequently self-removed his IV catheter. Intranasal (IN) dexmedetomidine was administered in a stepwise titration protocol (total dose: 100 μg). The patient transitioned from agitation to calm and entered sustained sleep within 1 h. The only hemodynamic alteration observed was mild bradycardia (heart rate: 58–65 beats/min), with no clinically significant respiratory depression occurring throughout the observation period. The Confusion Assessment Method (CAM) test result was negative on postoperative day 2, and the patient was discharged uneventfully on postoperative day 4. This case suggests that IN dexmedetomidine may offer a safe, non-invasive rescue option for established POD when IV access is unavailable.

## Introduction

Postoperative delirium (POD) is a common neuropsychiatric complication in elderly surgical patients, and it is associated with increased morbidity and mortality (1, 2). Managing established POD can be particularly challenging when agitated patients remove their IV lines. Intranasal (IN) drug delivery offers a non-invasive alternative, enabling access to the central nervous system via the olfactory and trigeminal pathways without hepatic first-pass metabolism (3). However, clinical evidence supporting the use of IN dexmedetomidine as a rescue therapy for established POD remains limited. In this report, we describe an elderly patient with POD who self-removed his IV catheter and was managed with a stepwise IN dexmedetomidine protocol.

### Case description

A 70-year-old man (height: 170 cm and weight: 65 kg) was admitted with a 20-day history of intermittent, painless, total gross hematuria throughout micturition. The hematuria had begun without an apparent cause, improved after intravenous therapy at an outside hospital, but subsequently recurred. He presented to our institution for further evaluation and was diagnosed with a bladder mass, benign prostatic hyperplasia, and a urinary tract infection. His medical history included hypertension lasting over 20 years, which was managed with regular antihypertensive medications and reported to be well controlled. Nine months earlier, he had been diagnosed with a cerebral infarction and underwent percutaneous cerebral angiography with intra-arterial thrombolysis; on the evening of this procedure, he reportedly developed agitation and restlessness, although the details of his diagnosis and management at that time were unavailable. He currently demonstrates no significant sequelae of prior cerebral infarction. There is no family history of dementia, neurodegenerative diseases, psychiatric illnesses, or other relevant hereditary conditions. He had completed 9 years of formal education and had smoked 10–20 cigarettes per day for more than 50 years, with no history of alcohol use.

On physical examination, his heart rate was 77 beats/min, blood pressure was 138/76 mmHg, and SpO_2_ was 96%. The neurological examination revealed intact cranial nerve function, preserved limb strength and sensation, and no focal neurological deficits. The patient was alert and fully oriented, with no subjective cognitive complaints; however, no formal baseline cognitive assessment was conducted preoperatively. A cranial CT scan revealed white matter degeneration with cerebral atrophy and an old infarct. Abdominal ultrasonography demonstrated bilateral renal cysts with focal wall calcifications, bilateral nephrolithiasis, and a solid posterior bladder wall lesion. The remainder of the workup was unremarkable. During the pre-anesthetic assessment, the patient was classified as ASA physical status III with a Mallampati score of II and a mouth opening of three finger-breadths. He was scheduled to undergo cystoscopy, transurethral electrocoagulation of the prostate, and a transrectal prostate biopsy under general anesthesia.

Anesthesia was induced with etomidate 20 mg, alfentanil 2 mg, and mivacurium 10 mg; a laryngeal mask airway was inserted. Anesthesia was maintained with remimazolam and remifentanil infusions, supplemented by low-concentration sevoflurane, with the bispectral index maintained between 40 and 60. Surgery concluded at 10:55 with stable hemodynamics. The patient achieved a Steward recovery score of 6 within 20 min and was returned to the ward.

At approximately 19:00 on the day of surgery, the patient developed agitation, disorganized speech, and insomnia. IV diazepam provided transient relief. On postoperative day 1, symptoms recurred with greater severity; the patient self-removed his IV catheter, and repeated diazepam produced no improvement. To exclude reversible and structural precipitants, a focused workup was undertaken: routine postoperative biochemistry showed a serum sodium of 138 mmol/L, potassium of 4.1 mmol/L, and chloride of 102 mmol/L, all within normal limits; oxygen saturation was maintained at 90%–99% and hemodynamics remained stable; the known urinary tract infection was already under active treatment; and no new focal neurological signs suggestive of acute stroke were detected. Anesthesiology consultation confirmed the presence of a POD according to the Diagnostic and Statistical Manual of Mental Disorders, Fifth Edition (DSM-5) criteria. Given the loss of IV access and poor cooperation, IN dexmedetomidine was selected.

**Figure 1:**
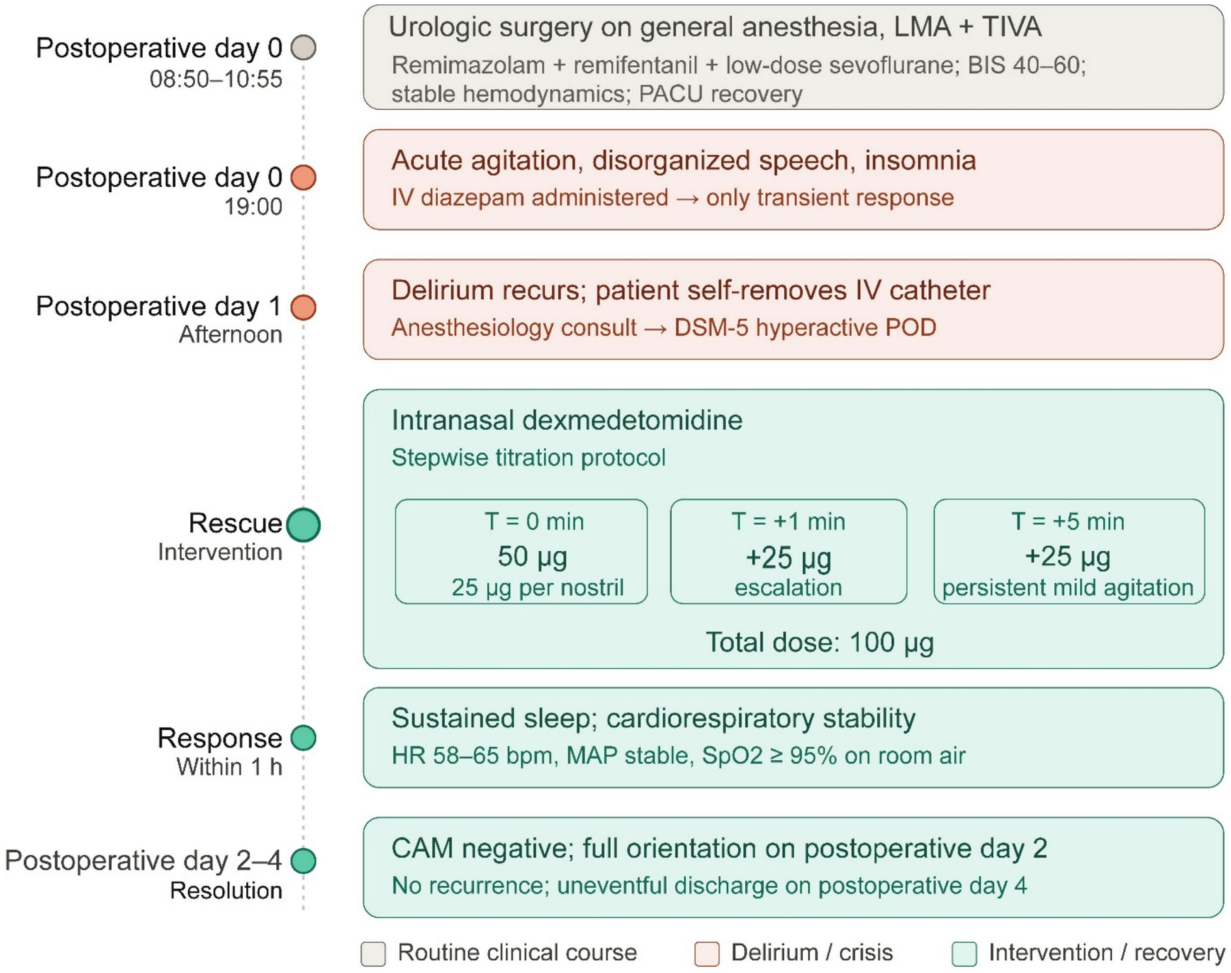
Clinical timeline of the perioperative course. BIS, bispectral index; CAM, confusion assessment method; DSM-5, diagnostic and statistical manual of mental disorders, fifth edition; HR, heart rate; IV, intravenous; LMA, laryngeal mask airway; MAP, mean arterial pressure; PACU, post-anesthesia care unit; POD, postoperative delirium; SpO_2_, peripheral oxygen saturation; TIVA, total intravenous anesthesia.

Following nasal priming, dexmedetomidine nasal spray (25 μg per spray; Jiangsu Hengrui Pharmaceuticals) was administered as one spray into each nostril. After 1 min, a third spray was delivered into one nostril; 5 min later, owing to persistent mild agitation, a fourth spray was administered into the contralateral nostril (total dose, 100 μg). The patient transitioned from agitation to calm and entered sustained sleep within 1 h. The vital signs remained stable with a heart rate ranging from 58 to 65 beats/min, systolic blood pressure between 130 and 155 mmHg, diastolic blood pressure between 70 and 95 mmHg, and SpO_2_ levels between 90% and 99%. No additional sedative or antipsychotic medications were administered. On postoperative day 2, the patient’s mental status improved markedly with full orientation restored, and the CAM assessment was negative. He remained free from delirium and was discharged on postoperative day 4 (Figure 1).

## Discussion

This patient carried multiple risk factors for POD, including advanced age, prior cerebral infarction, white matter degeneration, and a previous episode suggestive of delirium (4). Several aspects of anesthetic management warrant reflection: intraoperative analgesia relied on remifentanil without a structured postoperative analgesic plan, and sevoflurane inhalation may have contributed to the risk of POD (5). For elderly patients with cerebrovascular disease undergoing brief surgical procedures, a simplified anesthetic regimen comprising remimazolam-based induction, total intravenous anesthesia, and multimodal analgesia is strongly recommended.

The use of diazepam on two occasions without consulting an anesthesiologist is noteworthy. Benzodiazepines are generally known to exacerbate confusion and are therefore not recommended for routine management of delirium (6). Non-pharmacological interventions, including reorientation and sleep–wake cycle preservation, should accompany pharmacological management (6).

Intranasal dexmedetomidine was selected because of its non-invasive delivery and high level of patient acceptance. However, POD is inherently variable. Spontaneous resolution cannot be excluded, and dexmedetomidine may have primarily provided safe sedation rather than serving as a direct anti-delirium therapy. A recent case described a similar approach in the post-anesthesia care unit (7). The present case extends this experience to an older patient with a greater comorbidity burden managed in a ward setting.

Most clinical evidence for the use of intranasal dexmedetomidine derives from its application in perioperative settings rather than for treating established delirium. As a selective α2-adrenergic agonist, dexmedetomidine produces dose-dependent sedation that resembles natural non-rapid eye movement sleep and is largely free from clinically significant respiratory depression. When given intranasally, it bypasses hepatic first-pass metabolism (8). In the perioperative period, dexmedetomidine has been used extensively as an anxiolytic premedication and to prevent emergence agitation, particularly after sevoflurane-based anesthesia (9), and its administration has been reported to lower the incidence of postoperative delirium (10). In contrast, the evidence for the use of intranasal dexmedetomidine in treating established agitation or delirium is far more limited, with the available reports largely confined to pediatric patients or to the controlled setting of the post-anesthesia care unit; data regarding elderly patients with substantial comorbidities managed in general wards are scarce. The present case is therefore distinctive in three respects: dexmedetomidine was administered therapeutically rather than prophylactically; it was administered on the ward after intravenous access had been lost; and it was used in an elderly patient with significant cerebrovascular comorbidity.

In the present patient, stepwise administration of intranasal dexmedetomidine titrated to a total dose of 100 μg resolved the agitation within 1 h. The patient then experienced an uneventful recovery, with a negative CAM assessment result on postoperative day 2. This outcome highlights the potential of intranasal dexmedetomidine as a non-invasive rescue option for patients with established postoperative delirium when intravenous access cannot be maintained. However, further prospective studies are needed to confirm its efficacy and to define the optimal dosing regimen. In summary, for elderly patients with cerebrovascular disease who are at high risk of POD, perioperative strategies should optimize anesthesia, simplify medications, and enhance postoperative analgesia. Suspected POD requires early diagnosis and standardized treatment. Dexmedetomidine nasal spray provides a safe, non-invasive alternative when IV access is difficult or unavailable.

### Patient perspective

Consistent with the amnestic properties of delirium, the patient retained no recollection of the episode. On postoperative day 2, he reported awakening without distress and was notably surprised upon learning of his prior agitated state. He responded favorably when informed that intranasal administration had been used in lieu of intravenous injection, and expressed satisfaction with his overall care at the time of discharge.

## Data Availability

The original contributions presented in the study are included in the article/supplementary material, further inquiries can be directed to the corresponding author.
